# Diminished plasma levels of GPIb-*α* predict mortality in a prospective ARDS cohort

**DOI:** 10.3389/fmed.2026.1711751

**Published:** 2026-04-24

**Authors:** Alice Bernard, Lina Maria Serna-Higuita, Karina Althaus, Peter Rosenberger, Robin Vöhringer, Andreas Körner, Helene Haeberle, Claudia Eggstein, Tamam Bakchoul, Andreas Margraf, Christina Fodi, Alexander Zarbock, Michael Koeppen, Valbona Mirakaj

**Affiliations:** 1 Department of Anesthesiology and Intensive Care Medicine, University Hospital, Tuebingen, Germany; 2 Institute for Clinical Epidemiology and Applied Biostatistics (IKEaB), Tübingen, Germany; 3 Institute for Clinical and Experimental Transfusion Medicine, Medical Faculty, University Hospital Tübingen, Tübingen, Germany; 4 Department of Anaesthesiology, Intensive Care and Pain Medicine, University Hospital Münster, Münster, Germany; 5 Department of Anaesthesiology, Intensive Care and Pain Therapy, Goethe University Frankfurt, University Hospital Frankfurt, Frankfurt, Germany

**Keywords:** ARDS, biomarker, sepsis, GPIb-*α*, immunothrombosis, platelets

## Abstract

**Introduction:**

Activated immune cells can interact with platelets and trigger the coagulation cascade in response to inflammatory stimuli, resulting in the formation of microthrombi which help contain the spread of pathogens, but also contribute to organ dysfunction. Platelet glycoprotein receptor GPIb-*α* binds to von Willebrand factor and mediates cross-linking of platelets and thrombosis. In the multicenter Thilo trial, we measured plasma levels of GPIb-*α* to correlate our measurements with the clinical presentation of ARDS.

**Methods:**

GPIb-*α* plasma levels were measured on the first 5 days after study inclusion in 125 ARDS patients. Plasma levels of GPIb-*α* of survivors and non-survivors were compared; additionally, we issued a receiver operating characteristics (ROC) curve of GPIb-*α* levels and performed a survival analysis of patients stratified by their GPIb-*α* plasma levels. Multivariate Cox analysis was performed to identify risk factors for mortality.

**Results:**

Cox analysis showed that low GPIb-*α* levels were associated with higher mortality. In survivors, GPIb-*α* levels were significantly higher than in non-survivors. ROC analysis revealed an AUC value of 0.73 for the biomarker. For the Kaplan–Meier curve, patients with the lowest GPIb-*α* levels (0–130 ng/mL) had a mortality rate of 53%; in patients with higher levels (341–850 ng/mL), the mortality rate was 10%.

**Discussion:**

We are the first to report a role for platelet GPIb-*α* as a potential biomarker in ARDS. Lower GPIb-*α* plasma levels were associated with a higher mortality rate in ARDS. Diminished plasma levels might point to a higher consumption of the receptor, with an unfavorable effect on overall survival.

**Trial registration:**

EUDRA-CT: 2016-003168-37. Registered on 12/04/2017. ClinicalTrials.gov: NCT03111212. Registered on 04/06/2017.

## Introduction

The Acute Respiratory Distress Syndrome (ARDS) is a rapidly progressive form of organ dysfunction of the lung that accounts for 10% of all admissions to the ICU. Hallmarks of ARDS are life-threatening hypoxia, progressive self-propagating inflammation in the alveolar space and increased pulmonary vascular permeability (1). In ARDS, immunothrombosis adds to tissue destruction mediated by neutrophils (2–4). Platelets are key in mediating these effects, they can trigger and amplify the formation of neutrophil extracellular traps (NETs), participate in entrapment and eradication of pathogens and modulate the immune response through interaction with immune cells (5). Therefore, platelets are essential mediators between the immune system and hemostasis in ARDS and other diseases (6). In the early phase of acute inflammation, for instance, activated cellular factors, coagulation factors and other immune components can interact with platelets (7).

It is well known that the severity of ARDS is determined by the extent of inflammation within the lung and the alveolar space (8). Platelet surface receptors were examined in experimental ARDS in several investigations (9, 10). In this context, Burkard et al. could show that platelet glycoprotein VI (GPVI) positively influences neutrophil transmigration into the alveolar space, stabilizes platelet–neutrophil interaction, and adds to NET formation in the early phase of pulmonary inflammation (2).

The glycoprotein Ib alpha receptor (GPIb-*α*)—also known as CD42b—is a platelet surface receptor mediating platelet aggregation by binding to von Willebrand-factor (VWF) or other ligands. Together with glycoprotein Ib-*β* (GPIb-β) and glycoprotein IX (GPIX), it forms the GPIb-IX complex and promotes neutrophil–platelet interactions in sites of inflammation and vascular injury (11, 12), including polymicrobial sepsis (13), myocardial ischemia–reperfusion injury (14, 15), and venous shear stress (11). For COVID-19 infection, Li et al. were able to demonstrate that the spike protein of SARS-CoV-2 binds to GPIb-*α* to initiate platelet adhesion and consecutive immunothrombosis (16). Evidentially, immunothrombosis plays a pivotal role in causing tissue damage during lung inflammation and ARDS.

Known biomarkers for ARDS mainly include classical inflammatory biomarkers. Cytokines IL-6, IL-8, and IL-10, for instance, were associated with risk of ARDS development in traumatic brain injury (17); in COVID-19-ARDS, IL-6 might predict ARDS severity (18). Angiopoietin-2 has been shown to be prognostic for ARDS development in sepsis (19). In a multi-omics analysis, Liao et al. could work out that signaling pathways of—among others—TGF-*β* and IL-6 were associated with ARDS mortality, and that Angiopoietin-2 might be a potential biomarker for this purpose (20). Diminished levels of activated protein C have also been shown to be associated with increased mortality and a less favourable clinical course in acute lung injury; substitution of activated protein C in a clinical cohort with acute lung injury, however, did not improve patient outcome (21).

As a marker for endothelial dysfunction, VWF has been known to be associated with acute lung injury or ARDS, respectively, for over two decades (22, 23). VWF is a predictor for ARDS development in burn victims, for instance (24), and is involved in immunothrombosis in COVID-19 patients (25, 26).

As mentioned above, GPIb-*α* binds VWF. In more detail, platelet adhesion is mediated through the interaction of GPIb-*α* with VWF at the walls of injured vessels to initiate pathological thrombus formation. In shear stress, exposure of platelets to VWF causes shedding of GPIb-*α* from the platelet surface (27). Furthermore, *in vitro* and *in vivo* models showed that upon activation, platelets shed extracellular vesicles containing GPIb-*α*, which preferentially bind to monocytes; this mechanism leads to accumulation of GPIb-*α* on circulating monocytes in a murine model of lung injury, contributing to inflammatory damage (28). The shed form of GPIb-*α*—termed glycocalicin—is an established biomarker to determine platelet lifespan (29) and has been investigated as a diagnostic marker for diseases in which platelet dysfunction or damage plays a role (30–32); furthermore, glycocalicin is associated with conditions like shear stress. The function of GPIb-*α* shedding is still not clear, however, and a possible involvement in inflammation is conceivable (33).

We investigated the role of shed GPIb-*α* in plasma samples of patients with ARDS. Biomarkers correlated with mortality in ARDS could give further useful insight into underlying mechanisms orchestrating organ damage, and hint at crucial processes and even targets for future therapy options. We found that shed GPIb-*α* could be a promising biomarker for predicting survival of patients suffering from ARDS.

## Materials and methods

### Patient cohort

Patients were recruited between 2019 and 2021 in the context of the ThIlo trial, a randomized multicenter study exploring a potential therapeutic benefit of inhaled prostacyclin in ARDS (34, 35). The trial was approved by the Institutional Review Board of the Research Ethics Committee of the University of Tuebingen (899/2018AMG) and the corresponding ethical review boards of the participating centers, and by the Federal Institute for Drugs and Medical Devices (BfArM, EudraCT No. 2016–003168-37, registered on 12/04/2017). The ThIlo trial was registered at clinicaltrials.gov (NCT03111212, registered on 04/06/2017). In this trial, measurement of platelet activation markers was part of the trial protocol to determine their validity as potential biomarkers.

Our findings are therefore a *post hoc* analysis of a prospective ARDS cohort.

Out of the 144 patients whose data were analyzed in the primary analysis, plasma samples were available from 125 patients for biomarker analysis. Plasma samples were obtained once each day from day one to five. Patient data and follow-up—including monitoring for survival—were collected or performed, respectively, up to day 180 after study inclusion.

Diabetes mellitus was defined as a HbA1c level of 6.5% or higher. Pre-existing hypertension was defined as a systolic blood pressure of 130 mmHg or higher or of a diastolic blood pressure of higher than 80 mmHg as diagnosed by a physician at some point prior to hospital admission.

### Clinical complications

Sequential Organ Failure Assessment (SOFA) Score was used to evaluate quantity and extent of organ failure during ICU treatment (36).

Thrombocytopenia was defined as <150,000 cells/μL.

ARDS was defined, and severity categorized by the criteria of the Berlin definition (37).

Kidney dysfunction was defined according to the AKIN Classification of Acute Kidney Injury.

Thromboembolic complications were identified and defined, respectively, by their specific clinical, laboratory and imaging findings. Disseminated intravascular coagulopathy was identified by laboratory findings, clinical presentation and using DIC score (38). Pulmonary embolism, and embolism of other locations—portal vein, splenic vein, renal vein, jugular, intracranial—was identified by CT scan imaging findings. Thrombotic microangiopathy was identified by significant rise in laboratory findings such as D-dimers, and clinical presentation of organ-failure which could not be explained by other causes, and for which patients received specific and additional anti-coagulant treatment.

For treatment of ARDS, veno-venous extracorporeal membrane oxygenation (VV-ECMO) was initiated in some cases to ensure proper oxygenation, using an extracorporeal oxygenator (39).

### Sample preparation

Patient samples were retrieved in the mornings of each day from day 1 to 5 after study inclusion in an EDTA plasma tube. Immediately after collection, samples were centrifuged at 2,500 rpm for 10 min at room temperature. Afterwards, plasma was pipetted into cryotubes and stored at −80 °C.

### Measurement of GPIb-*α*

GPIb-*α* was measured using a commercially available sandwich ELISA (Thermo Fisher Scientific, #EH91RB, USA) according to the manufacturer’s instructions.

### Statistical analysis

Nominal variables were expressed as frequency and percentage. Quantitative variables were reported with mean and standard deviation or median and interquartile range, depending on the distribution of variables for each group. The assumption of normal distribution was evaluated graphically using histograms, Q-Q plots, box plots, skewness, and kurtosis. Bivariate analyses were performed using the appropriate chi-square test or Fisher’s exact test. Independent-sample *t*-tests were used to compare numerical variables that were normally distributed. In case of severe deviation from normal distribution even after log transformation, the non-parametric Mann–Whitney test was used.

Based on the GPIb-*α* levels, the patients were divided into quartiles. Kaplan–Meier survival analysis was used to analyze the association between different quartiles of GPIb-*α* levels and mortality. A Cox proportional hazard model was used to evaluate the association of the quartiles of GPIb-*α* levels and mortality. The results are reported as hazard ratios and 95% confidence intervals (CIs). The proportional hazard assumption of the Cox model was evaluated using Schoenfeld residuals. The goodness-of-fit of the final Cox model was checked using diagnostic plots based on the marginal and subject-specific residuals for the longitudinal outcome and the martingale and Cox–Snell residuals for the time-to-event outcome (40–45). A joint model (JM) technique was used to simultaneously analyze the correlation between GPIb-*α* over time and their influence on the time-to-event outcome (mortality). The JM was performed using the following three-step procedure: First, the individual trajectories of the GPIb-*α* obtained from the longitudinal evaluation were fitted using a linear mixed model, time was included as a fixed-effect variable, and random intercept and slope were included. In the second step, a Cox proportional model was used to evaluate the relationship between COVID-19, prostacyclin therapy and VV-ECMO. In the third step, the random effects (individual trajectories of GPIb-*α*) from the linear mixed model were incorporated as covariates in the survival model. The resulting joint model allowed for measuring the strength of the association between the dynamics of the GPIb-*α* biomarker and the hazard for mortality, including COVID-19, VV-ECMO, and prostacyclin therapy as a time-dependent variable.

Receiver operating characteristic (ROC) curves were drawn to measure GPIb-*α* in predicting mortality, and Youden index was used to determine a cut-off with the optimal sensitivity and specificity. Additionally, a linear mixed model was used to evaluate differences in platelet and GPIb-*α* concentration during the first 5 days in the intensive care unit (ICU); time, COVID-19, and therapy were used as fixed effects and subject as a random effect. Residuals were checked for normality and homogeneity of variance, and variables were log-transformed when needed.

Changes in GPIb-*α* (log-transformed) over time were evaluated using a linear mixed-effects modeling approach. All reported *p*-values were two-sided, and the significance level was ≤0.05. Bonferroni correction was performed to avoid type 1 error. All statistical analyses were performed using R statistical software version 4.1.

## Results

### Patient demographics

**Figure 1:**
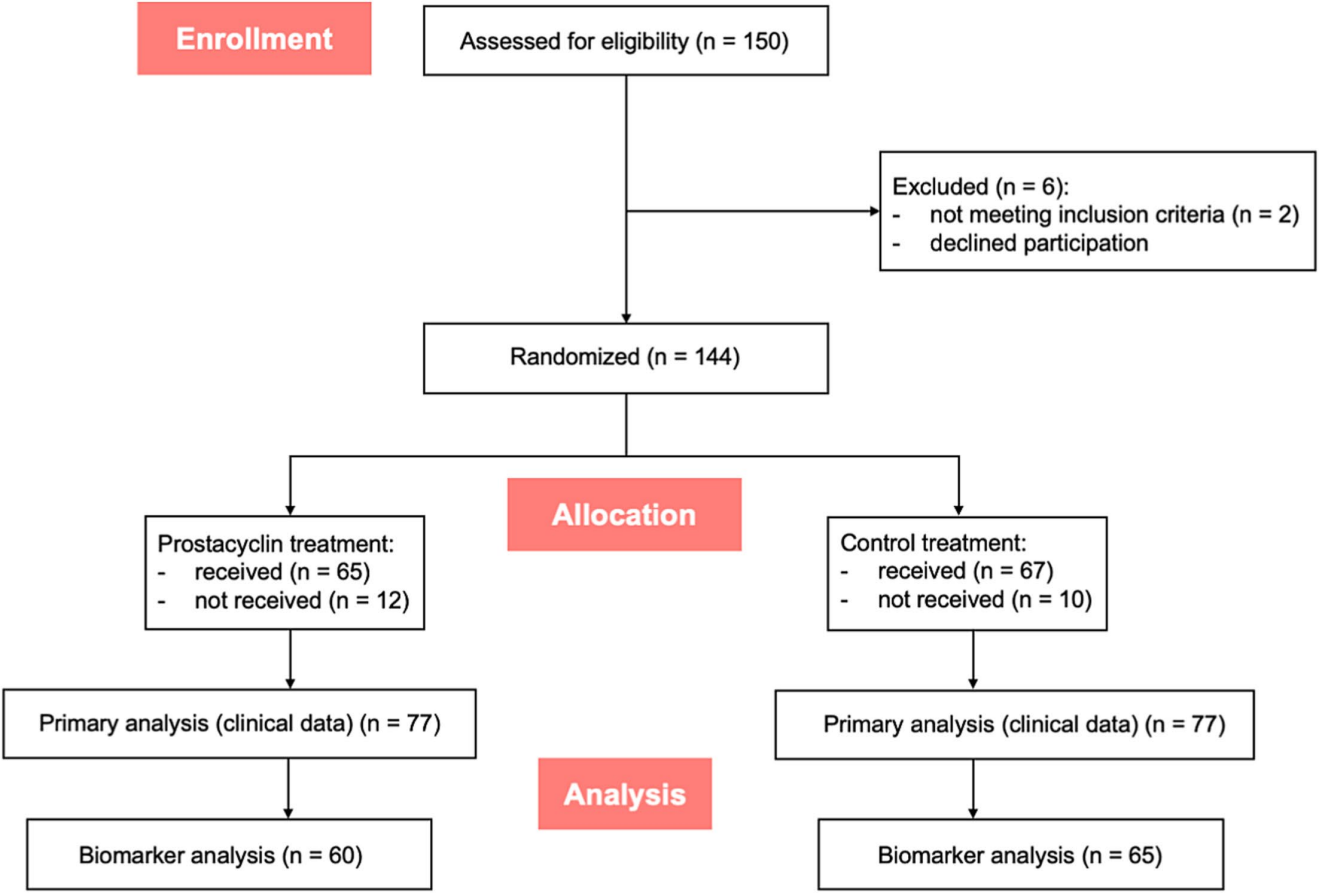
Flowchart of the recruitment and the follow-up analysis of the ThIlo trial.

Between July 2019 and May 2021, 150 patients were recruited within the ThIlo trial (37). Figure 1 shows a flowchart of the trial recruitment and the follow-up processes of the ThIlo trial, as well as the work-up for the biomarker analysis. Clinical data of 144 patients were available for the primary analysis (34), and plasma samples of 125 patients were available for measurement of GPIb-*α*. Missing plasma samples were mainly due to failure of retrieval.

Table 1 gives a summary of our patient cohort with available plasma samples for GPIb-*α* measurement stratified by survivors and non-survivors. Non-survivors were significantly older and showed higher SOFA scores at baseline. Thrombocytopenia was significantly more frequent in non-survivors, and absolute platelet count at baseline was significantly lower in non-survivors as well. Regarding pre-existing high blood pressure or diabetes, COVID-19 or VV-ECMO treatment, groups did not differ. Additionally, Supplementary Table 1 displays ventilation parameters at baseline for our cohort. Notably, most baseline parameters were not significantly different between survivors and non-survivors. Only the best Horovitz index (max. P_a_O_2_/F_i_O_2_) at baseline was significantly different, with higher values in the surviving group.

**Table 1:** Baseline characteristics of patients.

Parameter	Survivors (*N* = 84)	Non-survivors (*N* = 41)	Total (*N* = 125)	*p*-value
Age				
Mean (SD)	56.3 (14.8)	62.1 (13.4)	58.2 (14.6)	0.0335
Gender				
Male	58 (70.7%)	34 (85.0%)	92 (75.4%)	0.1351
Female	24 (29.3%)	6 (15.0%)	30 (24.6%)	
COVID				
No	21 (25.0%)	9 (22.0%)	30 (24.0%)	0.8794
Yes	63 (75.0%)	32 (78.0%)	95 (76.0%)	
Therapy				
NaCl	44 (52.4%)	21 (51.2%)	65 (52.0%)	0.999
Iloprost	40 (47.6%)	20 (48.8%)	60 (48.0%)	
Height				
Mean (SD)	173 (8.90)	176 (10.8)	174 (9.64)	0.155
Weight				
Mean (SD)	95.0 (22.7)	90.8 (17.0)	93.6 (21.0)	0.2533
Body mass index				
Mean (SD)	31.6 (7.13)	29.3 (5.56)	30.9 (6.72)	0.0542
Diabetes				
No	57 (72.2%)	28 (70.0%)	85 (71.4%)	0.9755
Yes	22 (27.8%)	12 (30.0%)	34 (28.6%)	
Hypertension				
Not existing	41 (51.9%)	18 (47.4%)	59 (50.4%)	0.7937
Known since > 1 year	38 (48.1%)	20 (52.6%)	58 (49.6%)	
VV-ECMO				
No	65 (77.4%)	25 (61.0%)	90 (72.0%)	0.0881
Yes	19 (22.6%)	16 (39.0%)	35 (28.0%)	
Transfusion of red blood cells				
No	64 (76.2%)	24 (58.5%)	88 (70.4%)	0.0686
Yes	20 (23.8%)	17 (41.5%)	37 (29.6%)	
Thrombocytopenia				
No	49 (58.3%)	13 (31.7%)	62 (49.6%)	0.009
Yes	35 (41.7%)	28 (68.3%)	63 (50.4%)	
Thrombocytes day 1				
Median (p25 – p75)	251 (178–332)	159 (135–215)	226 (152–302)	<0.001
Transfusion of platelets				
No	83 (98.8%)	38 (92.7%)	121 (96.8%)	0.198
Yes	1 (1.2%)	3 (7.3%)	4 (3.2%)	
SOFA baseline				
Mean (SD)	10.7 (3.24)	12.7 (3.17)	11.4 (3.33)	0.0019
Median [Min, Max]	11.0 [3.00, 18.0]	11.0 [8.00, 21.0]	11.0 [3.00, 21.0]	
Barthel baseline				
Median [p25 – p75]	0 (0, 0)	0 (0, 0)	0 (0, 0)	0.185

### Risk factors for mortality in our ARDS cohort

To evaluate risk factors for mortality in our cohort, we performed proportional Cox regression as a univariate model (Supplementary Table 2), and a multivariate model (Table 2). In the univariate analysis, parameters of organ dysfunction—represented by kidney dysfunction, SOFA score, and thrombocytopenia—as well as age were identified as significant factors. Neither prostacyclin treatment nor COVID-19 infection influenced mortality either way, which corroborates the results we previously published (34, 35). As mentioned above, GPIb-*α* was found to be of relevance in the formation of microthrombi caused by the SARS-CoV-2 virus (16), so we chose to measure GPIb-*α* plasma levels in our ARDS cohort, which included COVID-19 patients and non-COVID-ARDS patients (Table 1). GPIb-*α* levels at day 5 after study inclusion were identified as a significant factor influencing mortality in our cohort (Supplementary Table 2).

**Table 2:** Proportional Cox regression model for mortality, multivariable model.

Variable	HR	95% CI	*p* value	Chi Schoenfeld residuals	Harrell C
Age	1.03	1.00–1.06	0.049	0.386	0.696
GPIb-*α* day 5	0.49	0.29–0.81	0.005		
RBC transfusion	1.07	0.52–2.17	0.856		
Platelet transfusion	1.30	0.28–6.02	0.735		
aspirin therapy	1.18	0.55–2.53	0.676		
Prostacyclin treatment	0.98	0.92–1.04	0.953		
SOFA Score at baseline	1.07	0.96–1.19	0.208		

RBC: red blood cells.

In a multivariate Cox regression model (Table 2), only age and GPIb-*α* levels remained as significant influencing factors on mortality. Kidney dysfunction, as a well-known factor for mortality during ICU treatment (46), was not included in the multivariate model, as it—naturally—highly correlates with the SOFA score. We chose to use the SOFA score in this analysis to cover overall organ dysfunction.

### Lower plasma levels of GPIb-*α* predict mortality in our ARDS cohort

**Figure 2:**
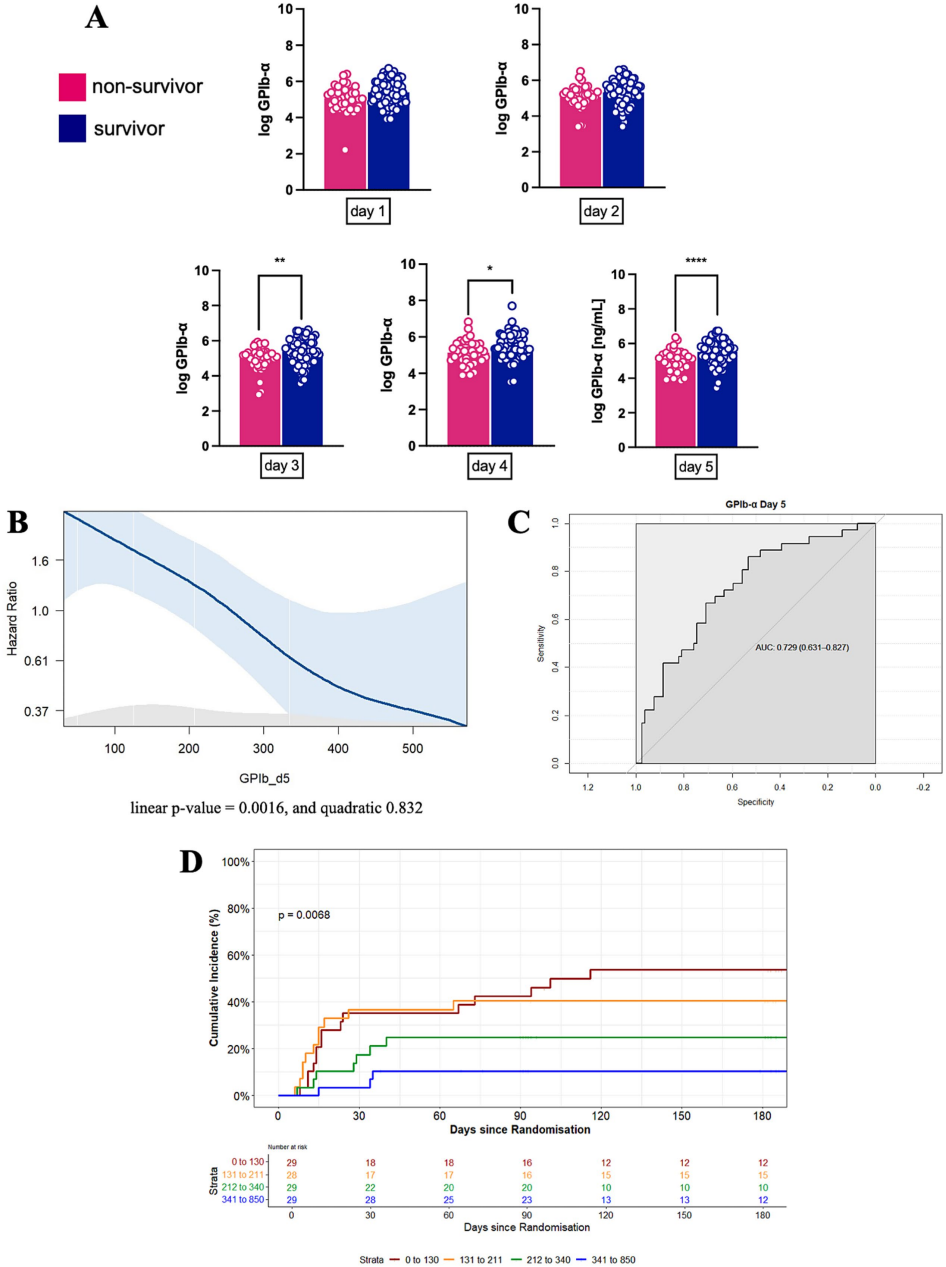
GPIb-*α* as a biomarker for mortality prediction in ARDS. **(A)** Comparison of GPIb-*α* plasma levels on day 1–5 after study inclusion between the survivors and non-survivors in our cohort (*n* = 84 for survivors; *n* = 41 for non-survivors; *p*-value as indicated). **(B)** Spline interpolation between GPIb-*α* levels and risk of death (linear *p*-value = 0.0016; quadratic = 0.832). **(C)** Receiver Operating Characteristic (ROC) curve of GPIb-*α* levels on day 5 after study inclusion. **(D)** Kaplan–Meier survival analysis up to day 180 after study inclusion stratified by quartiles for GPIb-*α* levels (Shown are SD or what exactly, *N* = 40 [non-survivors, day 1], *N* = 80 [survivors, day 1], *N* = 39 [non-survivors, day 2], *N* = 73 [survivors, day 2], *N* = 40 [non-survivors, day 3], *N* = 79 [survivors, day 3], *N* = 79 [non-survivors, day 4], *N* = 38 [survivors, day 4], *N* = 36 [non-survivors, day 5], *N* = 79 [survivors, day 5]).

Non-survivors had lower plasma levels of GPIb-*α* compared to patients still alive at day 180 after study inclusion: On day 3, 4 and 5 after study inclusion, GPIb-*α* levels were significantly lower in the non-survivors; on day 1 and 2, GPIb-*α* tended to be lower as well, though the difference did not meet significance (Figure 2A). As a sensitivity analysis, a joint model was carried out to evaluate the GPIb-*α* levels during the first 5 days. The joint model obtained by combining the linear mixed model and the survival submodel showed that GPIb-*α* levels were related to mortality (HR = 0.275, 95% CI 0.17–0.50, *p* < 0.001) (Supplementary Table 3). GPIb-*α* levels on day 5 correlated weakly with ARDS severity as represented by Horovitz index at study inclusion with a Pearson coefficient of 0.06 (Supplementary Table 4). Figure 2B visualizes the hazard ratio of GPIb-*α* and mortality via Splines interpolation, attributing plasma levels of about 100 ng/mL to an HR of over 1.6, while levels of about 300 ng/mL are attributed to an HR of about 0.6, overall showing an almost linear correlation. The Receiver Operating Characteristic (ROC) curve shows an AUC value of approximately 0.73 for GPIb-*α* on day 5 to discriminate between survivors and non-survivors (Figure 2C). Additionally, the Youden’s statistic determined an optimal threshold of 267 ng/mL of the GPIb-*α*, with a sensitivity of 53% and specificity of 86% for predicting mortality. For the Kaplan–Meier survival analysis (Figure 2D), GPIb-*α* levels at day 5 were sectioned into quartiles for risk stratification and reducing variability (0–130 ng/mL, 131–211 ng/mL, 212–340 ng/mL, 341–850 ng/mL). Here, incidence of mortality was highest in the lowest quartile (0–130 ng/mL), with a 53.6% mortality rate at day 180, while the highest quartile (341–850 ng/mL) had a mortality rate of only 10.3% (Table 3).

**Table 3:** Cumulative incidence mortality stratified by GPIb-*α* plasma levels on day 5.

GPIb-*α* (ng/mL)	1 month	2 months	3 months	6 months
0–125	35.1%	35.1%	42.3%	53.6%
126–207	36.6%	36.6%	40.4%	40.4%
208–335	17.3%	24.9%	24.9%	24.9%
336–849	3.4%	10.3%	10.3%	10.3%

**Figure 3:**
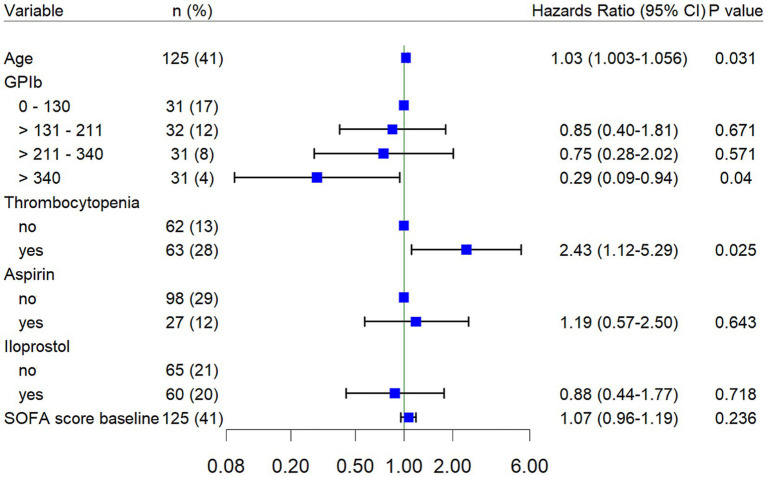
GPIb*α* levels were analyzed using quartile-based categorization due to the absence of validated clinical cut-off values or consensus thresholds. Quartiles were defined based on the distribution of GPIb-*α* levels in the study population, ensuring comparable numbers of patients per category. Hazard ratios (HRs) were estimated using Cox proportional hazards models, with the first quartile (lowest GPIb*α* levels) designated as the reference category and assigned an HR of 1. HRs for higher quartiles are expressed relative to this reference. Error bars indicate 95% confidence intervals.

Figure 3 shows a Forest plot of various factors influencing mortality in our cohort. The lowest quartile of GPIb-*α* levels (0–130 ng/mL) was assigned a HR of 1, and used as a benchmark for determining the HR of higher GPIb-*α* levels: Levels of 131–210 ng/mL have a HR of 0.85 (95% CI: 0.04–1.81, *p* = 0.671), levels of 211–340 ng/mL show a HR of 0.75 (95% CI: 0.28–2.02, *p* = 0.571) and levels higher than 340 ng/mL have a significantly reduced HR of 0.29 (0.09–0.94, *p* = 0.04).

### Thromboembolic events were associated with increased mortality in our cohort, and GPIb-*α* levels increase over time if no thromboembolic event has been recorded

For 117 patients, GPIb-*α* plasma levels were measured, and further clinical data was accessible to be analyzed for the occurrence of thromboembolic complications.

Supplementary Table 5 gives an overview of the type of complication recorded in the patients’ files. Cases classified as “suspected” were counted into the analysis as “positive” if symptoms, clinical course and laboratory findings were plausibly indicating the presence of thromboembolic complications (e.g., decrease in D-dimers, organ dysfunction, correlation with further diagnostic finding in CT scan or ultrasound diagnostic, fibrinolysis shutdown in elaborated coagulation testing).

In our cohort, 59 patients presented with at least one kind of thrombotic complication; out of these, seven patients experienced more than one kind of complication.

As seen in Supplementary Table 6, plasma levels of GPIb-*α* tended to be higher in patients not showing thromboembolic complications, with differences reaching statistical significance on day 4 (day 2 no longer met significance after Bonferroni correction). In a mixed model—as seen in Table 4—the mean GPIb-*α* value was lower in the group that experienced thromboembolic events than in the group without such complications, but this difference was not statistically significant (*β* = −0.54, *p* = 0.656). The longitudinal courses of GPIb-*α* over time (days 1 to 5) revealed no changes in the mean values of GPIb-*α* during the 5-day follow-up period (beta 0.015, *p* = 0.219). The group with thromboembolic events, however, compared to the group without thromboembolic events showed significantly greater daily reductions: (*β* = −0.082, *p* = 0.001), corresponding to a 7.8% daily reduction in GPIb-*α*.

**Table 4:** Mixed model (random intercept), outcome slope of GPIb-*α* (log transformed).

Parameter	*β*	95% CI	*P*-value
Intercept	5.310		
Thromboembolic event yes	−0.054	−0.294 to 0.184	0.656
Time	0.015	−0.009 to 0.041	0.219
Interaction	−0.082	−0.132 to −0.032	0.001

### Correlation of GPIb-*α* plasma levels with platelet count, platelet aggregation inhibitors, and antithrombotic therapy, and mortality

As GPIb-*α* is a platelet surface receptor primarily involved in platelet adhesion and platelet aggregation through binding of VWF (14), we investigated a possible connection between overall platelet count or thrombocytopenia over time (first 5 days), respectively, and drugs influencing platelet function (aspirin, clopidogrel), or coagulation (heparin, argatroban).

**Figure 4:**
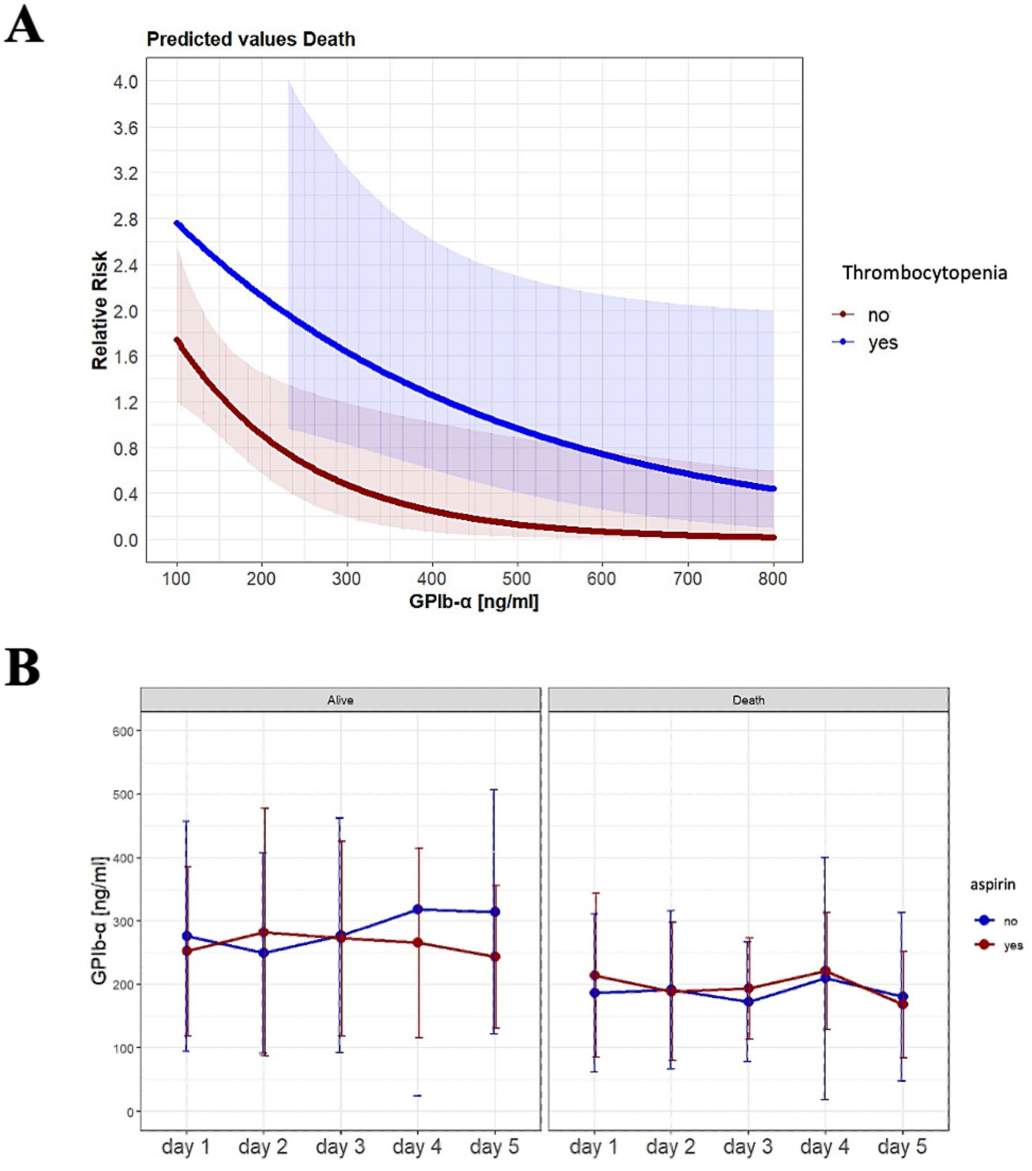
Interaction of GPIb-*α* with platelet count, platelet aggregation inhibitors, antithrombotic therapy, and blood transfusion: **(A)** Cox model for the relationship between GPIb-*α*, thrombocytopenia, and risk of death. **(B)** Mean values of GPIb-*α* stratified by therapy with aspirin and compared between survivors and non-survivors. (Shown are SD or what exactly, *N* = 40 [non-survivors, day 1], *N* = 80 [survivors, day 1], *N* = 39 [non-survivors, day 2], *N* = 73 [survivors, day 2], *N* = 40 [non-survivors, day 3], *N* = 79 [survivors, day 3], *N* = 79 [non-survivors, day 4], *N* = 38 [survivors, day 4], *N* = 36 [non-survivors, day 5], *N* = 79 [survivors, day 5]).

GPIb-*α* levels did not correlate with platelet numbers (repeated measure correlation *r* = 0.18, 95% CI 0.09–0.27) (Supplementary Figure 1). As seen in Table 1, incidence of thrombocytopenia at baseline was more frequent in non-survivors compared to non-survivors. A multivariable Cox regression model was used to investigate the impact of the relationship between GPIb-*α* at day 5 and mortality across patients with and without thrombocytopenia. The model included the following variables: GPIb-*α* at day 5, thrombocytopenia (yes/no) and the interaction term (Supplementary Table 7). There was no significant interaction between thrombocytopenia and GPIb-*α* levels at day 5 (Supplementary Table 7; Figure 4A). As seen before in Table 2 and Supplementary Table 2, respectively, treatment with aspirin did not influence risk of death. Additionally, occurrence of aspirin intake was not distributed significantly different in survivors and non-survivors (Figure 4B). Other platelet aggregation inhibitors—e.g. clopidogrel, ticagrelor—were not used in our cohort.

A multivariate Cox regression analysis for mortality shows that heparin (Supplementary Table 8, Supplementary Figure 2A) and argatroban (Supplementary Table 9, Supplementary Figure 2B) are unrelated to mortality.

### Correlation of platelet transfusions and GPIb-*α*

Platelet transfusions contain GPIb-*α* (47). Only four of our patients received platelet transfusions, showing significantly diminished levels of GPIb-*α* (Supplementary Table 10).

### Levels of GPIb-*α* do not differ between patients with COVID-19-ARDS and non-COVID-ARDS

Platelet activation and immunological thromboembolism are a key mechanism for damage mediated by SARS-CoV-2. Li et al. demonstrated how the virus activates platelets by binding its spike protein to GPIb-*α*—also termed CD42b—*ex vivo.* In our cohort, levels of GPIb-*α* though do not differ between patients with ARDS based on COVID-19, and ARDS of non-COVID origin (Supplementary Figure 3A), and the effect of COVID-19 infection on mortality was not affected by the levels of GPIb-*α* (Supplementary Table 11; Supplementary Figure 3B). As also seen in Table 2 and Supplementary Table 2, COVID-19 was not associated with a higher risk of death.

### GPIb-*α* levels are independent of VV-ECMO treatment

VV-ECMO therapy did not influence GPIb-*α* levels (Supplementary Figure 4), and ECMO therapy was not associated with an alteration in mortality (Supplementary Table 2).

## Discussion

We report here for the first time that levels of the platelet surface receptor GPIb-*α* correlate with ARDS mortality and might be a potential biomarker for ARDS disease severity. Through the binding of VWF, but also several other ligands, GPIb-*α* leads to platelet aggregation and immunothrombosis (14). In our ARDS cohort, we found that lower levels of GPIb-*α* in plasma samples are associated with increased mortality.

As mentioned above, the pathophysiology of ARDS is marked by loss of pulmonary barrier function, the infiltration of inflammatory cells into the alveolar space and hypoxia. Activated platelets are critically involved in the release of inflammatory mediators such as interleukin-1β, which reduces the endothelial barrier function enhancing fluid leakage and retention in the interstitial space (48). Platelets also orchestrate the cell–cell interactions of inflammatory cells attracting polymorphonuclear cells (PMN) to the lung, and PMNs are critical mediators of the pulmonary tissue injury in ARDS (2). The formation of microthrombi increases this tissue damage and forms the link between inflammation and hemostasis, a process termed immunothrombosis (7, 49). There is also reciprocal signaling between neutrophils and platelets that is critical for the activation of neutrophils in the pulmonary defense against invading bacteria, which is not present if platelets are depleted (50). Several studies have shown that platelet GPIb-*α* is essentially involved in these processes, and that it is an important regulator of platelet activation. As such, determining the levels of GPIb-*α* as a potential biomarker in the plasma could be a valuable tool in the diagnosis and management of ARDS patients.

Influencing the coagulation system is a potential target in ARDS treatment. In a Phase-III-trial, Dixon et al. found nebulized heparin might have beneficial effects on development of ARDS in at-risk patients and could—among other results—reduce lung injury (according to the Lung Injury Score by Murray). The authors attributed this to heparin-mediated reduction fibrin deposition, but no biomarker-based evaluation was performed in their cohort to corroborate their hypothesis (51); heparin, however, is known to have anti-inflammatory effects (52), and the authors could prove the safety of nebulized heparin in their trial (51).

As mentioned above, there are several biomarkers that were previously evaluated in ARDS patients, von Willebrand factor (VWF)—a GPIb-*α* ligand—being one of them. In more detail, Rubin et al. showed that elevated levels of VWF were present in patients that developed lung injury opposed to critically ill patients that did not (53); the involvement of VWF in inflammation is the subject of vast and yielding research, with convincing findings linking VWF to inflammatory processes and inflammatory damage (54, 55).

Along these findings, data from other trials corroborate the connection of VWF and inflammatory damage *in vivo*: An analysis of samples from the PROCESS trial showed that high levels of VWF were associated with an increased mortality in sepsis (56). Studies focusing on immunothrombosis in COVID-19 ARDS show that prothrombotic markers VWF (57) and the kallikrein/kinin system—as another part of the mechanisms—are increased and mark a less favorable outcome (58).

Selectins—a group of cell adhesion molecules—might also be of interest in this context, as they mediate the adhesion between leukocytes and endothelial cells, and P-Selectin is found on the surface of activated platelets, contributing to the adhesion of platelets to endothelial cells. A study on ARDS patients, however, revealed that only low levels of L-Selectin—found on leukocytes—were associated with a progression to ARDS in patients admitted to the ICU, while soluble P-Selectin—a potential marker on the surface of platelets—did not show a significant difference in this patient collective (59). In sepsis patients admitted to the ICU, elevated levels of soluble P-Selectin were associated with progression to sepsis with a predictive value of 0.78 (60). Both studies measured levels of soluble—i.e. shed—levels of the protein, and as mentioned above, P-Selectin is not an exclusive marker of platelets, but also present on endothelial cells. Therefore, this makes it impossible to attribute the measured injury to a specific cell type.

As of today, there are no studies which have evaluated the role of platelets or a specific platelet surface marker on the outcome of ARDS.

Our study shows several limitations that need to be considered. First, the plasma concentration of GPIb-*α* did not correlate with the platelet numbers in patients. One might think that this should be the case, since this receptor is expressed on the surface of platelets. The fact that we measured the plasma levels of the shed receptor, and not the platelet-bound form, might be the reason why GPIb-*α* levels did not correlate with platelet numbers, or thrombocytopenia. Yet, we hypothesize that it is the shedding of GPIb-*α* from the platelet surface that is protective or at least beneficial in ARDS. As survivors had higher plasma levels, it is conceivable that the lower levels might mark a consumption of GPIb-*α* during formation of microthrombi which increases disease severity and mortality in ARDS, even though further research is needed to further evaluate the causality of our findings. We can report, however, that decreases in GPIb-*α* over a five-day course were higher in patients experiencing at least one clinically apparent thromboembolic event, which might hint at a consumption of the biomarker.

We measured GPIb-*α* in our cohort—consisting of ARDS patients with both COVID-19-ARDS and Non-COVID-ARDS—as a possible marker for COVID-specific immunothrombosis. Interestingly, even though it has been shown that SARS-CoV-2 initiates immunothrombosis via binding to GPIb-*α*, plasma levels did not differ between patients with COVID-19 ARDS and ARDS of other origin, pointing at the existence of common mechanisms that uses GPIb-*α* during immunothrombosis. Levels of GPIb-*α* were also not influenced by VV-ECMO therapy, even though ECMO is known to cause platelet dysfunction.

Another limitation of our results certainly is the fact that the ThIlo trial was not intended to investigate immunothrombosis; anticoagulation protocols and therapy with platelet aggregation inhibitors was not standardized in our cohort.

Our data represent a *post hoc* analysis of a prospective ARDS cohort, not a targeted trial investigating GPIb-*α* in ARDS, and further prospective trials need to be designed to specifically investigate more deeply into the relationship between GPIb-*α* and ARDS course and outcome, respectively.

In this context, we need to acknowledge the fact that platelet function and coagulation is affected by many factors, the most prominent being medication such as aspirin or antithrombotic therapy, which many—or in case of the antithrombotic therapy: all—patients received. Aspirin, for instance, is discussed as reducing the risk of ARDS developments through the reduction of NET formation, and inhibition of ThromboxanA_2_ (61). In our cohort, there was no difference in mortality or GPIb-*α* levels in respect to aspirin intake, though, as mentioned above.

In summary, we could show that GPIb-*α* might hold the potential to be a predictor for mortality in ARDS, with lower levels bringing with it a higher risk of death. It is without question, though, that further research is needed to understand the mechanisms and possible intervention points for therapeutic options for immunothrombosis in ARDS.

## Data Availability

The raw data supporting the conclusions of this article will be made available by the authors, without undue reservation.

## Supplementary material

Supplementary Figure 1 Correlation between GPIb-*α* plasma levels and platelet count [repeated measures correlation *r* = 0.18 (0.09–0.27)].

Supplementary Figure 2 (A) Relationship between GPIb-*α*, heparin, and the risk of death. (B) Relationship between GPIb-*α*, argatroban, and the risk of death.

Supplementary Figure 3 (A) Survivors and non-survivors stratified by COVID-19-ARDS. (B) Relationship between GPIb-*α*, COVID-19 infection, and the risk of death. (Shown are SD or what exactly, *N* = 40 [non-survivors, day 1], *N* = 80 [survivors, day 1], *N* = 39 [non-survivors, day 2], *N* = 73 [survivors, day 2], *N* = 40 [non-survivors, day 3], *N* = 79 [survivors, day 3], *N* = 79 [non-survivors, day 4], *N* = 38 [survivors, day 4], *N* = 36 [non-survivors, day 5], *N* = 79 [survivors, day 5]).

Supplementary Figure 4 GPIb-*α* plasma levels of patients with and without VV-ECMO treatment.
