# Supplementary material for: Diminished plasma levels of GPIb-α predict mortality in a prospective ARDS cohort
Source: Front Med (Lausanne). 2026 Apr 24;13:1711751. doi: 10.3389/fmed.2026.1711751 (PMC13153104; doi:10.3389/fmed.2026.1711751)

**Supplemental Table 1:** Baseline ventilator parameters of patient cohort

|  | **Survivors (N=84)** | **Non-survivors (N=41)** | **Total (N=125)** | **P-value** |
| --- | --- | --- | --- | --- |
| **Max compliance** |  |  |  |  |
| Mean (SD) | 56.3 (21.6) | 58.8 (23.6) | 57.1 (22.2) | 0.6046 |
| **Min P_a_O/FiO_2_** |  |  |  |  |
| Mean (SD) | 159 (76.0) | 139 (60.6) | 152 (71.7) | 0.104 |
| **Max P_a_O_2_/FiO_2_** |  |  |  |  |
| Mean (SD) | 252 (92.5) | 208 (91.8) | 238 (94.2) | 0.013 |
| **Acidosis** |  |  |  |  |
| No | 48 (57.1%) | 20 (48.8%) | 68 (54.4%) | 0.4902 |
| Yes | 36 (42.9%) | 21 (51.2%) | 57 (45.6%) |  |
| **Min SpO_2_** |  |  |  |  |
| Median [p25, p75] | 94 [90, 99] | 93 [89, 95] | 93 [91, 96] | 0.569 |
| **Max SpO_2_** |  |  |  |  |
| Median [p25, p75] | 100 [98, 100] | 100 [99, 100] | 100 [98, 100] | 0.099 |
| **Min svO_2_** |  |  |  |  |
| Median [p25, p75] | 74.5 [68, 78] | 74.5 [73, 75] | 74.5 [70, 77] | 0.943 |
| **Max svO_2_** |  |  |  |  |
| Median [p25, p75] | 78 [69, 79] | 77.5 [77, 78] | 77.5 [76, 79] | 0.829 |
| **Invasive ventilation** |  |  |  |  |
| No | 8 (9.5%) | 1 (2.4%) | 9 (7.2%) | 0.2845 |
| Yes | 76 (90.5%) | 40 (97.6%) | 116 (92.8%) |  |
| **No-invasive ventilation** |  |  |  |  |
| No | 75 (89.3%) | 38 (92.7%) | 113 (90.4%) | 0.778 |
| Yes | 9 (10.7%) | 3 (7.3%) | 12 (9.6%) |  |
| **Prone position** |  |  |  |  |
| No | 22 (26.2%) | 8 (19.5%) | 30 (24.0%) | 0.55 |
| Yes | 62 (73.8%) | 33 (80.5%) | 95 (76.0%) |  |
| **Max Pmax** |  |  |  |  |
| Mean (SD) | 28.2 (6.16) | 28.4 (4.83) | 28.3 (5.74) | 0.8702 |
| **Max Pmean** |  |  |  |  |
| Mean (SD) | 19.7 (3.95) | 21.2 (5.65) | 20.2 (4.60) | 0.1432 |
| **Min PEEP** |  |  |  |  |
| Mean (SD) | 11.9 (3.51) | 12.0 (3.49) | 12.0 (3.49) | 0.8844 |
| **Max PEEP** |  |  |  |  |
| Mean (SD) | 13.6 (2.99) | 14.3 (3.31) | 13.8 (3.10) | 0.2527 |
| **Driving pressure at max Pmax** | |  |  |  |
| Mean (SD) | 12.9 (4.03) | 11.9 (4.91) | 12.6 (4.35) | 0.2696 |
| **Tracheotomy** |  |  |  |  |
| No | 84 (100%) | 41 (100%) | 125 (100%) | NA |
| Yes | 0 (0%) | 0 (0%) | 0 (0%) |  |
| **High frequency ventilation** |  |  |  |  |
| No | 84 (100%) | 41 (100%) | 125 (100%) | NA |
| Yes | 0 (0%) | 0 (0%) | 0 (0%) |  |

**Supplemental Table 2:** Proportional Cox regression model for mortality (univariate model)

|  | **HR** | **95% CI** | **P value** | **Schoenfeld residuals** |
| --- | --- | --- | --- | --- |
| Age | 1.03 | 1.002 – 1.05 | 0.030 | 0.27 |
| Gender Female | 0.56 | 0.23 – 1.33 | 0.188 | 0.28 |
| COVID-19 yes | 1.23 | 0.659 – 2.58 | 0.579 | 0.007 |
| Prostacyclin therapy | 0.99 | 0.53 – 1.82 | 0.966 | 0.61 |
| BMI | 0.96 | 0.91 – 1.01 | 0.121 | 0.110 |
| GPIb-α day 5 | 0.45 | 0.30 – 0.68 | <0.001 | 0.50 |
| Cell red transfusion | 1.77 | 0.95 – 3.29 | 0.072 | 0.20 |
| Platelet transfusion | 3.05 | 0.4 – 9.90 | 0.063 | 0.81 |
| Thrombocytopenia | 2.57 | 1.33 – 4.97 | 0.005 | 0.18 |
| Aspirin | 1.60 | 0.82 – 3.13 | 0.171 | 0.57 |
| Heparin | 0.99 | 0.53 – 1.84 | 0.988 | 0.58 |
| Argatroban | 0.96 | 0.50 – 1.81 | 0.891 | 0.90 |
| SOFA baseline | 1.13 | 1.03 – 1.24 | 0.006 | 0.34 |
| Kidney dysfunction | 3.63 | 1.98 – 6.67 | <0.001 | 0.40 |
| ECMO | 0.59 | 0.97 – 3.38 | 0.062 | 0.41 |

**Supplemental Table 3:** A joint model exploring mortality risk

| **Variable** | **β** | **SE** | **HR** | **CI 95%** | **P value** |
| --- | --- | --- | --- | --- | --- |
| Age | 0.032 | 0.013 | 1.03 | 1.005 – 1.06 | 0.016 |
| Transfusion RBC | 0.017 | 0.365 | 0.84 | 0.41 – 1.72 | 0.633 |
| Transfusion platelets | 0.147 | 0.755 | 1.15 | 0.26 – 5.08 | 0.846 |
| iloprost | 0.060 | 0.342 | 1.06 | 0.54 – 2.08 | 0.861 |
| GPIb-α | -1.259 | 0.273 | 0.28 | 0.17 – 0.49 | <0.001 |
| SOFA baseline | 0.096 | 0.055 | 1.10 | 0.98 – 1.23 | 0.084 |

**Supplemental Table 4:** Linear regression of GPIb-α on day 5 and min. P_a_/F_i_O2 at baseline

|  | **Beta** | **95% CI** | **P value** |
| --- | --- | --- | --- |
| Intercept | 13.1 |  |  |
| Log GPIb – α | 27.8 | 8.29 to 47.32 | 0.006 |

R square 0.0664, Pearson correlation 0.24

**Supplemental Table 5:** Type and frequency of thromboembolic events in our cohort.

| **Thrombemcolic complication** | **No. of cases** |
| --- | --- |
| Pulmonary embolism | 32 |
| Thrombembolic micoangiopathy | 30 |
| Disseminated intravascular coagulation | 12 |
| Jugular vein thrombosis | 3 |
| Portal vein thrombosis | 1 |
| Renal vein thrombosis | 1 |
| Splenic vein thrombosis | 1 |
| Deep vein thrombosis | 1 |
| Gastrointestinal embolism | 1 |
| Intracranial embolism | 1 |

**Supplemental Table 6:** GPIb-α levels stratified by thromboembolism

|  | **No Thromboembolism (N=67)** | **Thromboembolism (N=50)** | **Total (N=117)** | **P-value** |
| --- | --- | --- | --- | --- |
| **GPIb baselines** |  |  |  |  |
| [min, max] | [9.17, 829.04] | [49.9, 698.4] | [9.17, 829.0] | 0.3727 |
| Median [Q1, Q3] | 219.5 [127.5, 356.2] | 186.5 [137.4, 304.5] | 209.9 [134.2, 335.2] |  |
| N | 63 | 49 | 112 |  |
| **GPIb D1** |  |  |  |  |
| [min, max] | [29.87, 741.1] | [31.5, 664.5] | [29.9, 741.1] | 0.6908 |
| Median [Q1, Q3] | 213.3 [140.4, 292.6] | 193.3 [132.5, 276.6] | 211.3 [137.2, 286.9] |  |
| N | 62 | 47 | 109 |  |
| **GPIb D2** |  |  |  |  |
| [min, max] | [18.89, 704.90] | [21.9, 760.8] | [18.9, 760.8] | 0.0763 |
| Median [Q1, Q3] | 214.29 [162.38, 369.4] | 176.8 [120.1, 300.6] | 203.9 [132.8, 335.6] |  |
| N | 64 | 49 | 113 |  |
| **GPIb D3** |  |  |  |  |
| [min, max] | [33.7, 2204.26] | [34.8, 922.7] | [33.7, 2204.3] | 0.0482 |
| Median [Q1, Q3] | 268.8 [148.7, 395.3] | 192.5 [137.3, 297.3] | 208.9 [146.6, 358.1] |  |
| N | 65 | 46 | 111 |  |
| **GPIb D4** |  |  |  |  |
| [min, max] | [31.1, 848.8] | [41.61, 753.24] | [31.1, 848.8] | 0.0031 |
| Median [Q1, Q3] | 278.4 [167.7, 410.5] | 182.2 [96.4, 293.5] | 211.9 [131, 351.3] |  |
| N | 65 | 46 | 111 |  |
| **GPIb D5** | [9.17, 829.0] | [49.9, 698.4] | [9.2, 829] | 0.3727 |
| [min, max] | 219.5 [127.5, 356.2] | 186.5 [137.4, 304.5] | 209.9 [134.2, 335.2] |  |
| Median [Q1, Q3] | 63 | 49 | 112 |  |
| N |  |  |  |  |

**Supplemental Table 7:** Multivariate Cox regression model for mortality

| **Variable** | **HR** | **95% CI** | **P value** |
| --- | --- | --- | --- |
| GPIb-α day 5 | 0.99 | 0.98 – 0.999 | 0.011 |
| Thrombocytopenia | 1.003 | 0.29 – 3.43 | 0.99 |
| Thrombocytopenia:GPIb-α | 1.004 | 0.998 – 1.01 | 0.162 |

**Supplemental Table 8:** Proportional Cox regression model for mortality (multivariate model)

| **Variable** | **HR** | **95% CI** | **P value** |
| --- | --- | --- | --- |
| GPIb-α day 5 | 0.99 | 0.99 – 0.999 | 0.028 |
| Heparin | 1.12 | 0.36 – 3.47 | 0.849 |
| Heparin:GPIb-α | 0.998 | 0.99 – 1.004 | 0.542 |

**Supplemental Table 9:** Proportional Cox regression model for mortality (multivariate model)

| **Variable** | **HR** | **95% CI** | **P value** |
| --- | --- | --- | --- |
| GPIb-α day 5 | 0.99 | 0.98 – 0.999 | 0.015 |
| Argatroban | 0.65 | 0.20 – 2.10 | 0.473 |
| Argatroban: GPIb-α | 1.003 | 0.997 – 1.01 | 0.379 |

**Supplemental Table 10:** Comparison of GPIb-α plasma levels between patients having received no platelet transfusions (not) and platelet transfusions (yes)

|  | **not (N=121)** | **yes (N=4)** | **Overall (N=125)** | **P-value** |
| --- | --- | --- | --- | --- |
| **GPIb-α d1** |  |  |  |  |
| Mean (SD) | 250 (164) | 95.9 (46.7) | 246 (164) | 0.0109 |
| **GPIb-α d2** |  |  |  |  |
| Mean (SD) | 237 (153) | 88.4 (24.0) | 233 (153) | < 0.001 |
| **GPIb-α d3** |  |  |  |  |
| Mean (SD) | 249 (162) | 88.3 (65.8) | 243 (162) | 0.0092 |
| **GPIb-α d4** |  |  |  |  |
| Mean (SD) | 284 (252) | 131 (44.2) | 278 (249) | < 0.001 |
| **GPIb-α d5** |  |  |  |  |
| Mean (SD) | 269 (174) | 92.3 (55.8) | 263 (175) | 0.0021 |

**Supplemental Table 11:** Comparison of GPIb-α plasma levels between patients non-COVID-ARDS (no) and COVID-19-ARDS (yes)

|  | **no (N=65)** | **yes (N=60)** | **Overall (N=125)** | **P-value** |
| --- | --- | --- | --- | --- |
| **GPIb-α d1** |  |  |  |  |
| Mean (SD) | 218 (164) | 255 (163) | 246 (164) | 0.3004 |
| **GPIb-α d2** |  |  |  |  |
| Mean (SD) | 202 (159) | 243 (150) | 233 (153) | 0.2463 |
| **GPIb-α d3** |  |  |  |  |
| Mean (SD) | 229 (156) | 248 (164) | 243 (162) | 0.5819 |
| **GPIb-α d4** |  |  |  |  |
| Mean (SD) | 243 (154) | 289 (272) | 278 (249) | 0.2631 |
| **GPIb-α d5** |  |  |  |  |
| Mean (SD) | 266 (190) | 262 (171) | 263 (175) | 0.9063 |


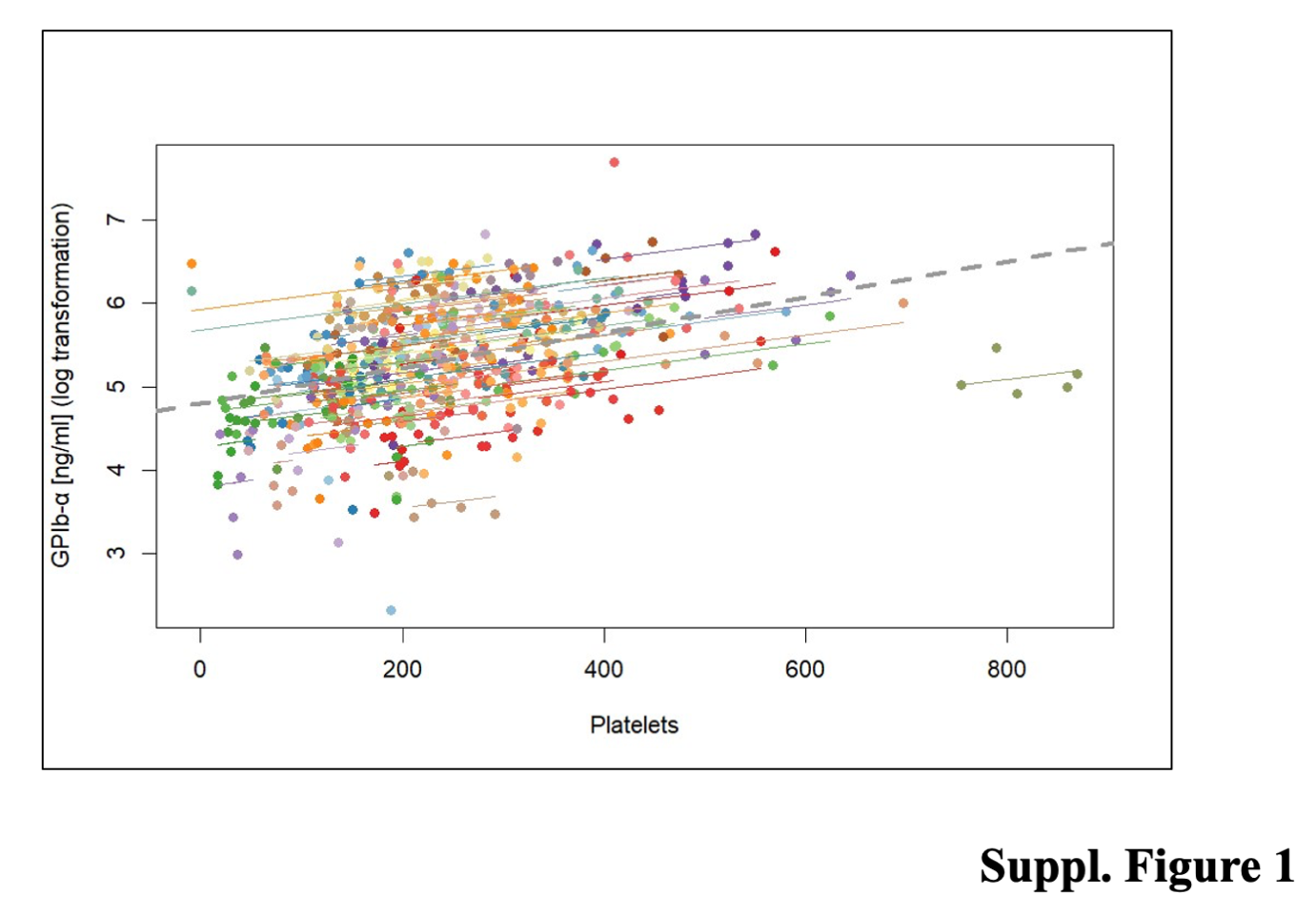


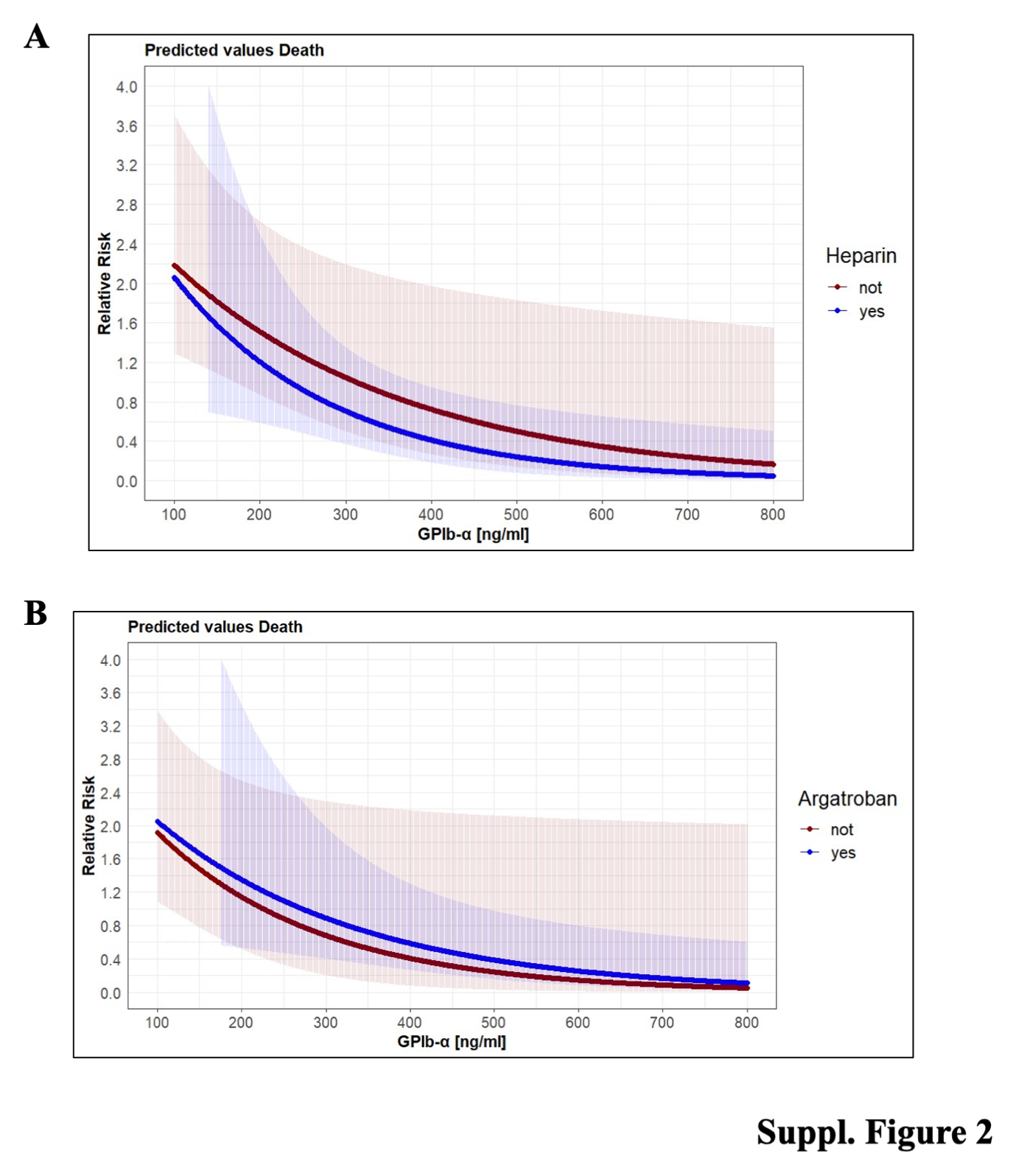


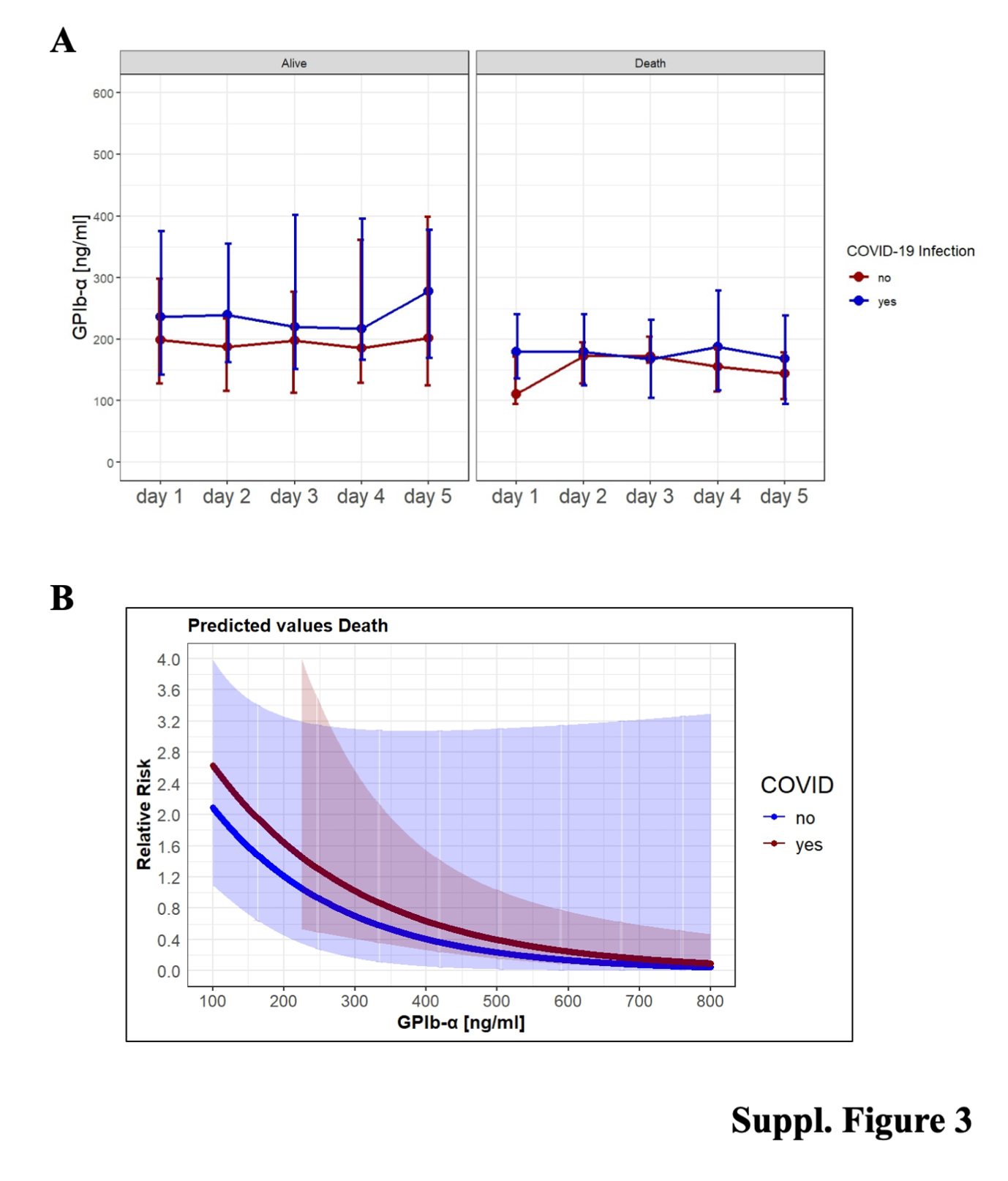


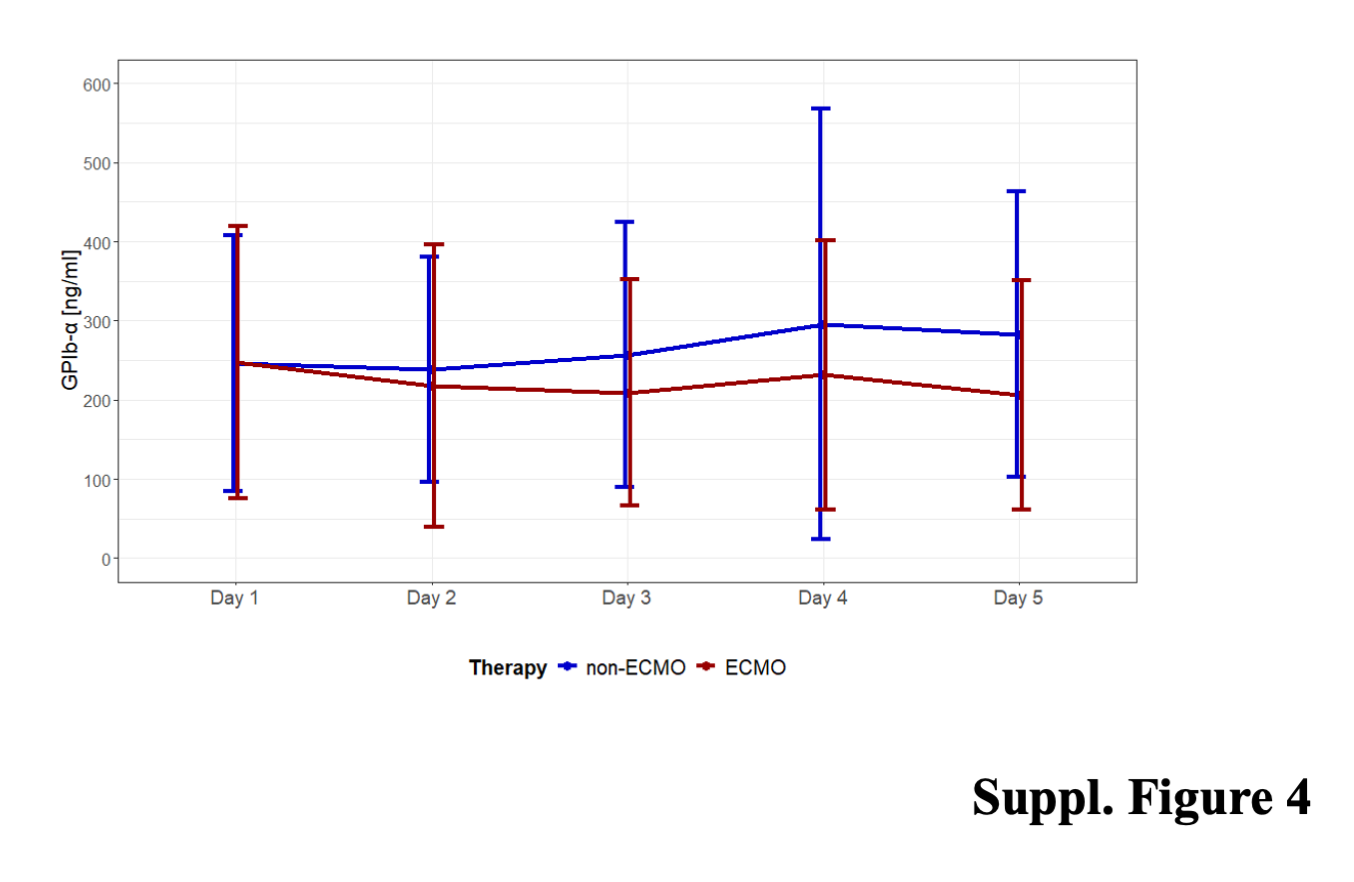

Supplement: Supplementary Figure 1 — Correlation between GPIb-α plasma levels and platelet count [repeated measures correlation r = 0.18 (0.09–0.27)]. [file Data_Sheet_1.DOCX]
